# Influence of Geographical Origin on Isotopic and Elemental Compositions of Pork Meat

**DOI:** 10.3390/foods12234271

**Published:** 2023-11-26

**Authors:** Adriana Dehelean, Ioana Feher, Puscas Romulus, Dana Alina Magdas, Florina-Dorina Covaciu, Angela Maria Kasza, Victor Curean, Gabriela Cristea

**Affiliations:** 1 National Institute for Research and Development of Isotopic and Molecular Technologies, 67-103 Donat Street, 400293 Cluj-Napoca, Romania; adriana.dehelean@itim-cj.ro (A.D.); ioana.feher@itim-cj.ro (I.F.); romulus.puscas@itim-cj.ro (P.R.); alina.magdas@itim-cj.ro (D.A.M.); florina.covaciu@itim-cj.ro (F.-D.C.); angela.kasza@itim-cj.ro (A.M.K.); 2 Faculty of Pharmacy, “Iuliu Hatieganu” University of Medicine and Pharmacy, 400012 Cluj-Napoca, Romania; curean_victor@yahoo.com

**Keywords:** pork meat, geographical origin, IRMS, ICP-MS, chemometrics

## Abstract

Pigs are a primary source of meat, accounting for over 30% of global consumption. Consumers’ preferences are determined by health considerations, paying more attention to foodstuffs quality, animal welfare, place of origin, and swine feeding regime, and being willing to pay a higher price for a product from a certain geographical region. In this study, the isotopic fingerprints (δ^2^H, δ^18^O, and δ^13^C) and 29 elements of loin pork meat samples were corroborated with chemometric methods to obtain the most important variables that could classify the samples’ geographical origin. δ^2^H and δ^18^O values ranged from −71.0 to −21.2‰, and from −9.3 to −2.8‰, respectively. The contents of macro- and micro-essential elements are presented in the following order: K > Na > Mg > Ca > Zn > Fe > Cu > Cr. The LDA model assigned in the initial classification showed 91.4% separation of samples, while for the cross-validation procedure, a percentage of 90% was obtained. δ^2^H, K, Rb, and Pd were identified as the most representative parameters to differentiate the pork meat samples coming from Romania vs. those from abroad. The mean values of metal concentrations were used to estimate the potential health risks associated with the consumption of pork meat The results showed that none of the analyzed metals (As, Cd, Sn, Pb, Cu, and Zn) pose a carcinogenic risk.

## 1. Introduction

The global population has increased, leading to a rising demand for meat. But, worldwide, the trends are not equally distributed. Consumption of beef and lamb is decreasing, while people are eating more and more pork and poultry [1]. Due to their biological qualities, pigs represent one of the species majorly involved in meeting the population’s meat needs, accounting for over 30% of world consumption. In Romania, pork is a traditional product, with a relatively constant consumption throughout the year, slightly higher during the Christmas period, with the annual mean consumption per capita being around 36 kg [2]. Pork represents about 45% of the total meat production in Romania, in 2022 reaching nearly 342,000 metric tons. Pig breeding, under the conditions of our country, is a very important economic branch [3]. Nevertheless, pig meat imports have exceeded 300,000 metric tons annually in recent years [2,3].

The pork industry of the European Union has been affected in recent years by a Russian trade embargo, the COVID-19 pandemic, and African swine fever [4]. In addition, in 2022, the prices of pork meat increased due to high energy costs and high costs of cereals [5]. In this context, it has reached the point that Romania imports almost 90% of the required pork, thus affecting the local producers [3]. Also, consumer preferences and habits will be determined by health considerations, paying more attention to foodstuffs quality, animal welfare, place of origin, and swine feeding regime.

Isotope Ratio Mass Spectrometry (IRMS), together with Inductively Coupled Plasma Mass Spectrometry (ICP-MS), represents efficient analytical techniques that trace the geographical origin of different food products, especially if these techniques are combined with statistical methods. Such results are reported for wine [6,7], milk and dairy products [8,9], eggs [10], honey [11], olive oil [12,13], and saffron [14]. Just as each person has a unique fingerprint, each plant and each animal have unique characteristics linked to their place of origin. The isotopic signatures of ^2^H and ^18^O are related to the corresponding isotopic values of drinking water and water present in animal feed, which show geographical variability [15]. The ^13^C isotopic fingerprint of meat reflects the feeding regime of the swine, based on C3 (cereals (wheat, barley, oats, and rye) and potatoes, etc.) or C4 (maize) plants. Furthermore, each geographical region presents its specific geological and pedological features supported by elemental contents [16]. A certain geographical area can influence the elemental nutrients in a natural way, on the soil–water–plant–animal chain, conducting to a food product enriched in that/those specific element/elements [17].

In addition, heavy metal contamination of foodstuffs, particularly meat, represents an increasing concern, and several studies [18,19,20,21] have been conducted to assess the health risks associated with heavy metal exposure through meat consumption. The estimated daily intake (EDI), target hazard quotient (THQ), hazard index (HI), and target cancer risk (TR) via meat intake were used to compute the heavy metal concentrations and assess the likely health risks posed to consumers.

In this context, the aims of our study were as follows: (i) Highlighting the ^2^H, ^18^O, and ^13^C isotopic signature of investigated pork meat samples as a function of geographical origin; (ii) Recording their elemental concentrations; (iii) Performing Principal Component Analysis (PCA) to assess if samples from different geographical origins offer the potential to cluster into separate groups, and applying Linear Discriminant Analysis (LDA) to identify and point out the best differentiation markers related to a sample’s place of origin; (iv) Conducting human health risk assessment through pork meat consumption.

## 2. Materials and Methods

### 2.1. Sampling

A total of 70 pork meat samples (loin) were investigated in order to determine their isotopic and elemental profiles. From these, 37 samples were labeled “Romania”, coming from different regions of the country. The rest of the 33 pork meat samples originated from abroad, being either bought from the Romanian market as imported goods or brought by colleagues who are away at conferences or on holidays in places such as Germany (n = 5), Spain (n = 25), and Hungary (n = 3), respectively. All samples (n = 70) were placed in plastic bags and stored in the fridge (not more than 24 h) until later preparation for stable isotope analysis and elemental fingerprint determinations were conducted.

### 2.2. Sample Preparation for Analysis

#### 2.2.1. Preparation for Stable Isotope Analysis

Samples preparation started with water extraction from each meat sample using a cryogenic distillation process under vacuum [22]. This process fulfilled two important requirements: (i) The extraction to be without isotopic fractionation; (ii) The amount of extracted water sufficient for subsequent isotopic analysis.

Once the cryogenic distillation was finished, the following data were obtained: (i) The total water amount which was contained in the meat sample; (ii) The meat sample completely dry. Then, from the extracted water, the isotopic signatures of ^2^H and ^18^O were determined. The dried meat sample was homogenized and crushed using a bill mill (MillMix 20, Domel, Železniki, Slovenia). The lipid content can affect the δ^13^C value, leading to wrong interpretations of the isotopic compositions. The ^13^C isotopic value from the “bulk” meat samples can be lower than that from the defatted sample as a consequence of the isotopic fractionation that occurred during lipid synthesis in plant and animal tissues. The solution to this issue consists of the chemical extraction of lipids from the respective matrix and conducting ^13^C stable isotope analysis on the protein fraction sample. During the lipid removal process, 0.5 g of dried meat, petroleum ether, and acetone were used. The resulting defatted meat sample was dried for 72 h in a Nabertherm oven (Lilienthal, Germany) at 60 °C. In the next stage, the defatted meat sample was subjected to dry combustion at 550 °C in oxygen excess for 3 h. The obtained CO_2_ was purified from other combustion gases by cryogenic separation and then measured by Isotope Ratio Mass Spectrometry (IRMS).

The equipment used for determining the ^2^H and ^18^O isotopic fingerprints of meat water was a liquid-water isotope analyzer (DLT—100, Los Gatos Research, San Jose, CA, USA), and for ^13^CO_2_, an isotope ratio mass spectrometer (Delta V Advantage, Thermo Scientific, Waltham, MA, USA) in line with a dual inlet system. The isotopic compositions (signature or fingerprint) are expressed in terms of delta values (δ) per mill (‰) vs. international standards, according to Equation (1) [23]:

(1) $$\delta^{i}X=\left(\frac{R_{\mathrm{sample}}}{R_{\mathrm{standard}}}-1\right)\times 1000,$$

where i represents the mass number of the heavier isotope of the element X (^2^H, ^18^O, ^13^C), R_sample_ is the isotope number ratio of a sample (^2^H/^1^H; ^18^O/^16^O; ^13^C/^12^C), and R_standard_ is that of the international standard (Vienna Standard Mean Ocean Water, V-SMOW, for δ^2^H and δ^18^O, and Vienna Pee Dee Belemnite, V-PDB, for δ^13^C). The standard deviation was ±1.0‰ for δ^2^H determinations and ±0.3‰ for δ^18^O, while for δ^13^C measurements it was ±0.3‰.

#### 2.2.2. Elemental Profile Analysis

The elemental concentrations (Na, Mg, Ca, K, Li, B, V, Cr, Mn, Fe, Co, Ni, Cu, Zn, As, Rb, Sr, Mo, Pd, Cd, In, Sn, Sb, Ba, La, Ce, Gd, Tl, and Pb) were analyzed using Flame Atomic Absorption Spectroscopy (FAAS) and ICP-MS methods. Prior to analysis, the pork meat samples were subjected to microwave-assisted nitric acid and hydrogen peroxide digestion. For sample mineralization, a microwave digester (Speed ENTRY by Berghof^®^) was used. In short, 500 mg of each sample (fresh weight) was accurately weighed directly into a PTFE digestion vessel, followed by the addition of 7 mL HNO_3_ (60% *v*/*v*) and 1 mL H_2_O_2_ (30% *v*/*v*). The microwave system was set to increase fromthe room temperature to 50 °C in 2 min and hold for 5 min; then from 50 to 75 °C in 2 min and hold for 15 min; from this temperature to 190 °C in 5 min and hold for 20 min; and finally, from 190 to 75 °C in 5 min and hold for 10 min. The digested solutions were left to cool to room temperature and then diluted with ultrapure water (resistivity 18 MΩ cm^−1^, Millipore, Bedford, MA, USA water purification system) to a final volume of 50 mL. The sample digestion of the blank solutions and certified reference material was made using the same preparation steps. The method’s accuracy was checked by using NCS ZC85006 as standard reference material. For elemental analysis, ICP-MS (Elan DRC (e), Perkin Elmer SCIEX^®^) and AAS (ContrAA 800D, Analytik Jena, Jena, Germany) were used. Certified multi-element solutions of 10 µg/mL (Ce, Dy, Er, Eu, Gd, Ho, La, Lu, Nd, Pr, Sm, Sc, Tb, Th, Tm, Y, and Yb, PerkinElmer Pure Plus, Billerica, MA, USA), 10 µg/mL Ag, Al, As, Ba, Be, Bi, Ca, Cd, Co, Cr, Cs, Cu, Fe, Ga, In, K, Li, Mg, Mn, Na, Ni, Pb, Rb, Se, Sr, Tl, U, V, and Zn (10 µg/mL, PerkinElmer Pure Plus, Billerica, MA, USA), and Au, Hf, Ir, Pd, Pt, Rh, Ru, Sb, Sn, and Te (10 mg/L, PerkinElmer Pure Plus, Billerica, MA, USA) were used for the standard stock solutions preparation, by dissolving the multi-element solutions with ultrapure water. For the calibration curve, the working solutions of a specific concentration and volume were prepared by diluting the stock solution.

#### 2.2.3. Statistical Analysis

The date on the isotopic and elemental content (δ^13^C, δ^18^O, δ^2^H, Mg, Ca, Na, K, Li, B, V, Cr, Mn, Fe, Co, Ni, Cu, Zn, As, Rb, Sr, Mo, Pd, Cd, In, Sn, Sb, Ba, La, Ce, Gd, Tl, and Pb) contained in the 70 meat samples were arranged as a matrix, in which the rows corresponded to the samples and the columns to the measured variables (X_70×32_). Different chemometric methods, such as analysis of variance (ANOVA), principal component analysis (PCA), and linear discriminant analysis (LDA), were used for the statistical interpretation of experimental data. ANOVA test was performed in order to find any differences in the compositions of investigated variables among meat samples from different countries. The data matrix was evaluated using two chemometric methods (PCA and LDA). Both PCA and LDA are linear transformation techniques used for dimensionality reduction. However, PCA is an unsupervised method, and LDA is a supervised one due to the fact that a dependent variable (DV) is considered and predefined with specific codes corresponding to each investigated class, making LDA a supervised model. PCA is a chemometric technique used for primary multidimensional evaluation of the data set, as well as to reduce the data dimensionality with minimal loss of useful information. It transforms the original data matrix into a product of two matrices, one containing information about the samples (score matrix) and the other containing information about the variables (loading matrix) [24]. LDA is a supervised method that aims to find a linear combination of features that best separates the classes in the dataset. It is a powerful tool for feature extraction and dimensionality reduction, which can be used to identify the most important variables that contribute to the separation of the classes [25]. All statistical tests were performed using SPSS v.20 (IBM, New York, NY, USA).

#### 2.2.4. Health Risk Assessment

Using the recommendations of the US EPA (United States Environmental Protection Agency) [26,27], the non-cancer risks related to the intake of As, Cd, and Pb-contaminated pork meat samples among the population were determined based on the estimated daily intake (EDI), targeted hazard quotient (THQ), and hazard index (HI), using the following formulas:

(2) $$\mathrm{EDI}=\frac{\left(C\times {\mathrm{IR}}_{d}\right)}{\mathrm{BW}},$$

(3) $$\mathrm{THQ}=\frac{\left(\mathrm{ED}\times C\times {\mathrm{IR}}_{d}\times \mathrm{EF}\right)}{\left(R_{f}D\times \mathrm{BW}\times \mathrm{AT}\right)}\times 10^{-3},$$

(4) HI=∑HQ,

where C is the metal concentration in the pork meat samples (mg/kg ww); IR_d_ represents the daily ingestion rate (g/day), corresponding to each country [28]; BW is the body weight (70 kg); ED is the exposure duration; EF represents the exposure frequency (365 days/year); R_f_D (μg/kg bw/day) is an oral reference dose of the metals that have no harmful effect during a lifetime; and AT represents the average exposure time (70 years).

If HI < 1, it means that the exposed population was assumed to be safe [29]. When HI ≥ 1, there is a moderate or high risk for adverse effects on humans. In our study, HI was calculated by the sum of the HQ(As), HQ(Cd), HQ(Pb), HQ(Sn), HQ(Cu), and HQ(Zn) because people suffer combined effects from exposure to several contaminants [30].

In addition, to indicate the carcinogenic risk of metals in meat samples, the target cancer risk was used using Equation (5) [31]:

(5) $$\mathrm{TR}=\frac{\mathrm{ED}\times \mathrm{EF}\times {\mathrm{IR}}_{d}\times C\times {\mathrm{CPS}}_{o}}{\mathrm{ABW}\times \mathrm{AT}}\times 10^{-3},$$

where TR represents the target cancer risk; CPS_o_ is the carcinogenic potency slope, oral (mg/kg bw/day); ABW is the average body weight (70 kg); and AT represents the average lifetime (70 years). The CPS_o_ values [31,32] are 1.5 mg/kg/day for As, 0.38 mg/kg/day for Cd, 0.0085 mg/kg/day for Pb, 1.5 mg/kg/day for Cu, and 0.3 mg/kg/day for Zn, respectively.

## 3. Results and Discussion

### 3.1. Isotopic Fingerprint of Hydrogen and Oxygen

**Figure 1:**
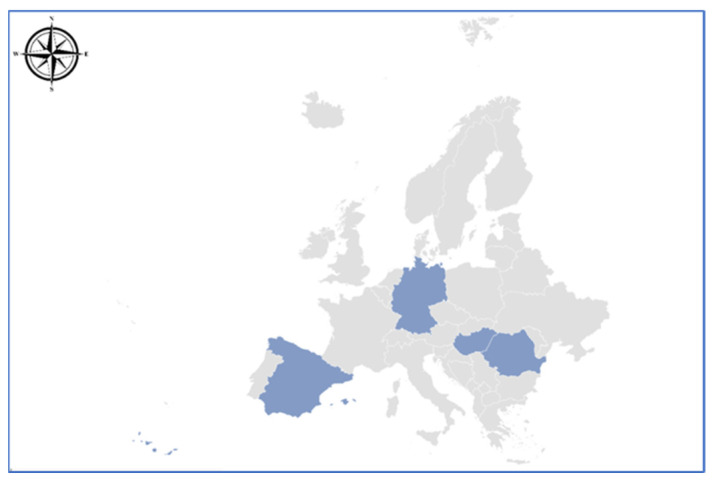
Map of the origin of meat samples as written on the labels.

To emphasize samples coming from different geographical regions, the isotopic compositions of hydrogen and oxygen are used. The basic principle behind the method consists of the fact that the isotopic signatures of ^2^H and ^18^O vary as a function of mean annual precipitation, distance from the sea, altitude, and ambient temperature [33,34]. Thus, δ^2^H and δ^18^O values of meat water from investigated samples decreased in order as follows: δ_Spain_ > δ_Germany_ > δ_Hungary_ > δ_Romania_ (Figure 1).

**Figure 2:**
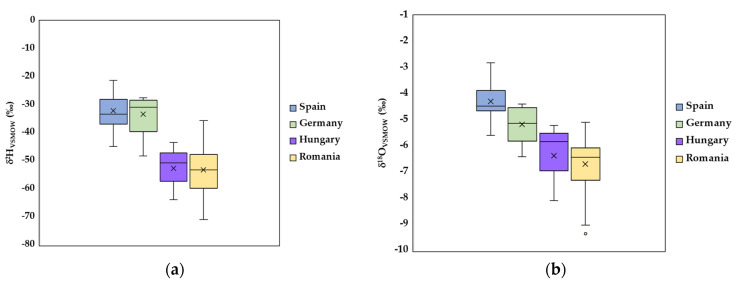
Box diagrams of δ^2^H (**a**) and δ^18^O (**b**) for the pork meat samples from Spain, Germany, Hungary, and Romania. The line across the boxes represents the median. Whiskers indicate the higher and lower values in the entire data range.

As expected, the higher isotopic values of δ^2^H and δ^18^O were recorded for samples labeled “Spain”, ranging from −44.8 to −21.2‰ for hydrogen and from −5.6 to −2.8‰ for oxygen, respectively (Figure 2). Spain is characterized by a Mediterranean climate on the south and east coast, a continental climate in central inland regions, and oceanic in north and northwest areas. It is known that the δ^2^H and δ^18^O values of precipitation decrease from low-latitude, low-elevation, and coastal regions to inland areas of high-latitude and mountainous regions [15]. Thus, the higher temperature in Spain compared to other countries, from where the investigated samples come from, led to intensive processes of plant transpiration and evaporation and subsequently to increased ^2^H and ^18^O isotope fingerprints of studied pork loin samples [9].

For Romanian pork meat samples, the isotopic composition of ^2^H ranged from −71.0 to −35.7‰, while those of ^18^O varied between −9.3 and −5.1‰. These results are not surprising, with Romania having a temperate–continental climate with moderate features characteristic of Central Europe, with distinct seasons. Following the hydrological cycle, the isotopic values (^2^H and ^18^O) of precipitation that fall in a certain area will be transferred to the drinking water from the respective location, which will be offered to animals, and then the isotopic signature of extracted water from animal tissue will be linked to that region [15,34]. Thus, lower values were recorded for Romanian meat samples collected during the cold season.

Samples labeled “Germany” had isotopic values in the range of −48.4 to −27.4‰ for δ^2^H, and from −6.4 to −4.4‰ for δ^18^O (mean of −5.2‰), respectively. These values are in the range of those reported previously [35] (δ^18^O varying from −8.0 and −6.0‰) and [36] (mean value of δ^2^H = −36.3‰, and mean value of δ^18^O = −4.1‰) for pork samples originating from Northern Germany, and also overlapped by another meat sample from Germany analyzed in our previous study [2].

Meat samples from Hungary presented similar values to those from Romania, these two countries being neighbors. Some Romanian samples come from Timisoara County, located in the western part of Romania, near the Hungarian border. As reported [37,38], more solid geographic differentiation of foodstuff samples using isotope and elemental profiles can be obtained by comparing products originating from regions far away from each other, whereas correct attribution is usually limited if production areas are geographically close to each other.

These results are also supported by results from the ANOVA test, with δ^2^H and δ^18^O displaying statistically significant parameters (*p* = 0.001 for both δ^2^H and δ^18^O values).

### 3.2. Isotopic Fingerprint of Carbon

In nature, a plant’s ^13^C isotope fingerprint depends on the photosynthetic pathway, C3 or C4. The majority of plant species on Earth use C3 photosynthesis (or the Calvin cycle), in which the first compound produced contains three carbon atoms. For these plants, *δ*^13^C values range between −30 and −23‰ (most vegetables, fruits, and cereals) [39]. In the C4 pathway (or Hatch–Slack cycle), a four-carbon compound is produced, and the isotopic signature of ^13^C is higher, varying from −14 to −12‰ [39]. The best-known examples from the C4 category are maize, sugarcane, sorghum, and millet. If maize (Zea mays) is introduced into an animal’s diet, the ^13^C isotopic results of meat will increase. Through the feeding regime, the animal will consume different plants, each of them having a specific isotopic signature, and the ^13^C value of the animal body will reflect the diet. Subsequently, the isotopic composition of meat samples will provide data regarding the proportion of C3 and C4 plants offered in the feeding process.

**Figure 3:**
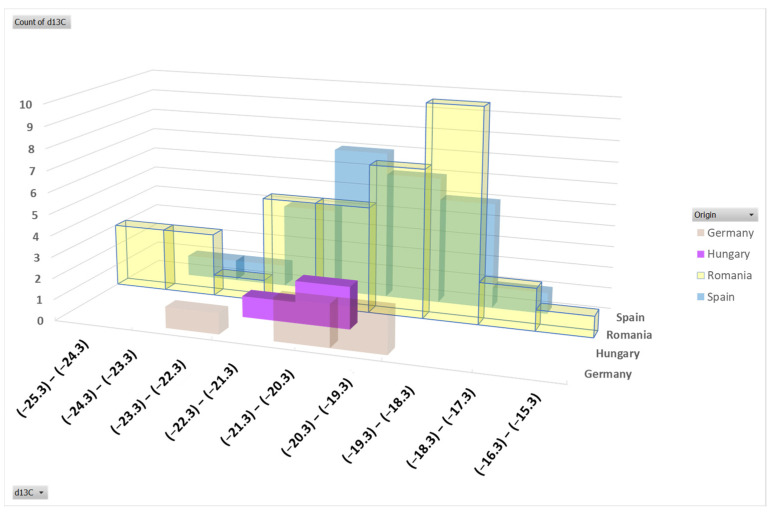
Frequency histogram based on the ^13^C isotopic composition of meat samples.

As can be observed in Figure 3, the δ^13^C range of variation for all meat samples is between −25.3‰ and −15.8‰ (mean value −20.6‰), both ends of the range belonging to samples coming from Romania. The mean values assigned to samples from Germany and Hungary were almost identical (−21.14 vs. −21.10‰), proving a combined diet formed from C3 and C4 plants and a similar way of feeding. The average value of δ^13^C for samples from Spain (−20.46‰) and Romania (−20.61‰) were much closer, demonstrating a higher proportion of corn in swine diet. The results for Spanish samples are similar to those previously published (−21.4‰) [40] and a little higher than those reported for Spanish conventional pork meat samples, which varied from −23.0 to −22.3‰ (mean value −22.6‰) [41].

Among the ^13^C signature of the Romanian data set, there are 12 samples with higher values, ranging from −19 to −15.8‰. The higher value was recorded for a sample from the northern part of the country, sampling in the cold season, demonstrating a feeding regime rich in maize. Usually, the proportion of corn in the feed increases progressively over autumn and winter until the swine are slaughtered, generally before Christmas. A corn-based diet is an old tradition in Romania [2], especially in Transylvania (the central and northwestern part of Romania); consumers prefer this specific taste of pork meat, given by a feeding regime enriched in corn. These isotopic values are in range with those previously published by our group [2,22]. Thus, the importance of meat authenticity with respect to geographical origin and breeding practices has increased because traditions related to a specific region always played a significant role in consumers’ choice of food.

### 3.3. Elemental Content

The concentrations of macro, micro, and trace elements in 70 pork meat samples with different geographical origins (Romania, Germany, Spain, and Hungary, respectively) are reported as mean values on a fresh weight basis in Table 1.

The contents of macro minerals (K, Na, Mg, and Ca) and micro essential elements (Fe, Cu, Zn, and Cr) obtained in this study showed the following order: K > Na > Mg > Ca > Zn > Fe > Cu > Cr. The same decreasing order was obtained for meat samples from China [42], Korea [43], Serbia [44], and Romania [22]. For our investigated samples, in terms of the concentration, K and Na were the most abundant elements, varying between 2.61 g/kg and 9.05 g/kg (K), 0.32 mg/kg and 0.92 g/kg (Na) (Spain); 3.11 g/kg and 4.79 g/kg (K), 0.33 g/kg and 0.64 g/kg (Na) (Germany); 4.24 g/kg and 5.21 g/kg (K), 0.49 g/kg and 0.63 g/kg (Na) (Hungary); 0.94 g/kg and 5.34 g/kg (K), 0.31 g/kg and 1.00 g/kg (Na) (Romania), respectively. In studies from other countries, K and Na concentrations were reported in the ranges of 3.60–4.43 g/kg and 0.36–0.41 g/kg (Korea) [43]; 2.6–4.44g/kg for K (Croatia) [45]; 15.05–14.37 and 1.34–1.43 g/kg (China) [42]; and 2.87–3.68 g/kg and 0.71–0.83 g/kg (South Africa) [46], respectively.

Macro elements are essential for various physiological functions in the body, including blood clotting [47], osmotic pressure [48], acid–base balance [49], muscle contraction [47], bone development [47], enzymatic activities [50], and hemoglobin synthesis [51]. The ratio of Na to K in any food item is an important factor to consider. High Na and low K intake can contribute to a greater prevalence of hypertension, a condition characterized by high blood pressure [52]. Several studies have shown that the Na/K ratio significantly affects hypertension prevalence and blood pressure [53,54,55]. In fact, a balanced Na to K ratio needs to be considered to prevent diet-induced secondary hypertension as a risk factor for cardiovascular disease [54]. The Na/K ratio in our body should be less than one. In the present study, the ratio of Na/K in pork meat was less than one, with values of 0.122 (Spain), 0.106 (Germany), 0.116 (Hungary), and 0.331 (Romania), respectively. This suggests that consuming the investigated pork meat can be beneficial for human health and may play a role in managing high blood pressure.

Mean concentrations of macro and micro essential elements (Table 1) were determined in the following ranges: Mg (0.14–0.71 g/kg), Ca (0.01–0.15 g/kg), Fe (1.91–17.87 mg/kg), Cu (0.12–1.88 mg/kg), Zn (3.26–17.33 mg/kg), and Cr (0.20–1.08 mg/kg) for Spain; Mg (0.22–0.27 g/kg), Ca (0.03–0.10 g/kg), Fe (4.85–10.80 mg/kg), Cu (0.65–1.37 mg/kg), Zn (5.25–10.03 mg/kg), and Cr (0.25–0.97 mg/kg) for Germany; Mg (0.25–0.29 g/kg), Ca (0.05–0.08 g/kg), Fe (3.30–19.19 mg/kg) Cu (0.38–1.07 mg/kg), Zn (5.37–9.33 mg/kg), and Cr (0.21–0.52 mg/kg) for Hungary; and Mg (0.16–0.32 g/kg), Ca (0.003–0.62 g/kg), Fe (1.55–22.06 mg/kg) Cu (0.28–2.27 mg/kg), Zn (3.77–19.94 mg/kg), and Cr (0.17–0.90 mg/kg) for Romania.

The World Health Organization (WHO) has classified Mn and Ni as probable essential elements for humans [56]. In the present study, the concentration ranges of these elements were 0.05–0.21 mg/kg (Mn) and 0.03–0.80 mg/kg (Ni) for Spain; 0.08–0.12 mg/kg (Mn) and 0.06–2.32 mg/kg (Ni) for Germany; 0.05–0.11 mg/kg (Mn) and 0.08–0.13 mg/kg (Ni) for Hungary; and 0.04–0.56 mg/kg (Mn) and 0.02–0.26 mg/kg (Ni) for Romania, respectively.

In the literature [57], trace elements, such as Li, V, Co, and Mo, are considered nontoxic elements, having mean concentrations less than 0.05 mg/kg in our study. In contrast, some trace elements, such as As, Cd, Sn, Tl, and Pb, are toxic due to their adverse effects on humans [58]. The mean concentrations of these elements were determined in the following ranges: Li: 0.01–0.09 mg/kg, V: 0.01–0.08 mg/kg, Co: 0.002–0.02 mg/kg, Mo: 0.005–0.08 mg/kg, As: 0.002–0.10 mg/kg, Cd: 0.0003–0.09 mg/kg, Sn: 0.01–1.05 mg/kg, Tl: 0.0001–0.08 mg/kg, and Pb: 0.02–0.17 mg/kg (Spain); Li: 0.01–0.04 mg/kg, V: 0.02–0.10 mg/kg, Co: 0.002–0.03 mg/kg, Mo: 0.004–0.09 mg/kg, As: 0.003–0.10 mg/kg, Cd: 0.0004–0.06 mg/kg, Sn: 0.05–0.39 mg/kg, Tl: 0.0003–0.07 mg/kg, and Pb: 0.02–0.11 mg/kg (Germany); Li: 0.04–0.05 mg/kg, V: 0.01–0.08 mg/kg, Co: 0.004–0.02 mg/kg, Mo: 0.01–0.04 mg/kg, As: 0.02–0.09 mg/kg, Cd: 0.0001–0.06 mg/kg, Sn: 0.06–0.12 mg/kg, Tl: 0.001–0.05 mg/kg, and Pb: 0.04–0.13 mg/kg (Hungary); and Li: 0.001–0.08 mg/kg, V: 0.01–0.09 mg/kg, Co: 0.0002–0.11 mg/kg, Mo: 0.001–0.09 mg/kg, As: 0.001–0.11 mg/kg, Cd: 0.0001–0.09 mg/kg, Sn: 0.01–0.17 mg/kg, Tl: 0.0002–0.10 mg/kg, and Pb: 0.005–0.24 mg/kg (Romania).

### 3.4. Chemometric Analysis (PCA and LDA)

**Figure 4:**
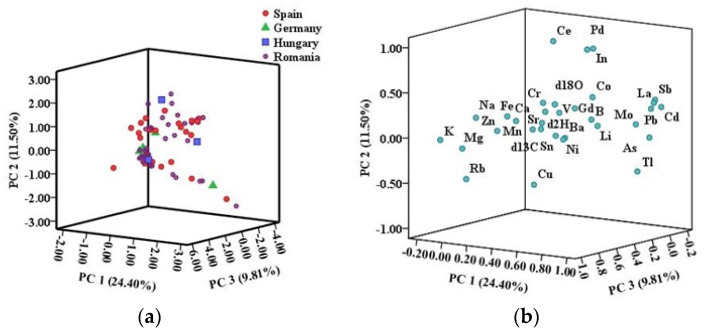
(**a**) Score plot of meat samples from different countries (Spain, Germany, Hungary, and Romania, respectively) using the first three principal components (PC1, PC2, and PC3) obtained after applying PCA to the entire isotopic and elemental data set X_70×32_. (**b**) Score plot of analyzed variables obtained using the first three principal components, explaining a total variance of 45.7%.

Firstly, PCA was performed to assess if samples from different geographical origins offer the potential to cluster into separate groups. The score plot of the first three principal components is indicated in Figure 4a. It can be observed that a perfect separation could not be obtained. The samples from Spain and Hungary were mainly located at positive values of PC1 scores, while the samples from Germany and Romania were placed at negative values for most samples. Another important representation resulting from PCA is the loadings plot (Figure 4b), where each analyzed element receives a coefficient correlated to its impact upon the sample set. Thus, the parameters with higher influence have higher coefficients and are situated far from the origin. In contrast, the variables with lower coefficients have a slighter effect and are much closer to the origin. All the parameters are grouped within principal components (PCs), the first PCs being the most important. Usually, only the first two or three components, which have eigenvalues higher than 1, are used for further interpretations. In the scatter plot in Figure 4a, the three components retained explained the 45.7% variability. PC1 explained the 24.4% of the variability based on As, Mo, Cd, Sb, La, and Pb having a positive contribution on PC1, and PC2 explained the additional 11.5% of variability where Ce, In, and Pd had a positive correlation on PC2. The third principal component, PC3, contributed to 9.8% of the variance based on K, Na, and Mg and could represent the geological influence upon sample distribution.

**Figure 5:**
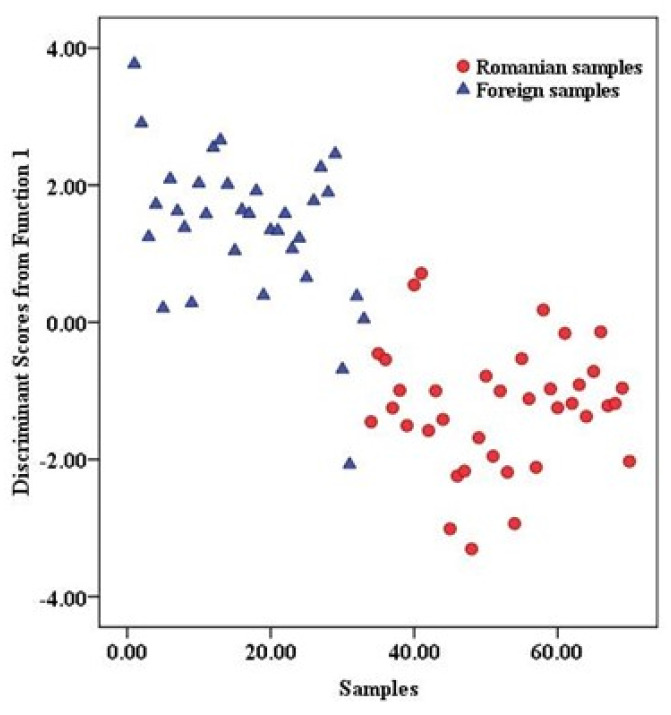
Meat samples separation, according to provenance (Romania or foreign origin), obtained using LDA.

An LDA approach was used to identify potential markers for differentiation for the pork meat samples from Romania and those from abroad. The “leave-one-out” cross-validation method was performed to evaluate the performance of this model. Because two classes (Romania vs. abroad) were compared, the discrimination was made based on one discriminant function (DF1) (Figure 5). By applying the LDA model, the obtained separation in the initial classification was 91.4%, while for the cross-validation procedure, a percentage of 90% was obtained (Table 2). The most representative markers were identified: δ^2^H, K, Rb, and Pd. The coefficients from the discrimination function of these significant parameters were 0.957 (δ^2^H), 0.662 (K), −0.714 (Rb), and −0.495 (Pd). LDA results show that four pork meat samples from a foreign group were assigned to the group from Romania, and three samples from the Romanian group were predicted as samples from abroad.

It is not surprising to obtain a ^2^H isotope fingerprint as a significant discrimination parameter, δ^2^H being a recognized marker for geographical origin identification [59,60]. These results are supported by another previously published study from our team [61], in which δ^2^H and Ti represented statistically significant predictors for geographical origin discrimination of Transylvanian (Romania) carrot samples.

Rb ions act like K ions for living organisms [62], and the body treating Rb ions like K ions. Rubidium (Rb) was also recorded as a differentiation predictor for geographical origin in other published studies for rice [63], sesame seeds [64], typical Italian alpine cheeses [65], beef samples [66,67], and saffron [59]. It seems that soil acidity, a lower pH of the soil, influences Rb absorption by plants [64]. In our previous study regarding pork meat authenticity [2], Rb was obtained as a key variable to discriminate investigated samples.

The fingerprints of dried beef from different countries were investigated, obtaining ^104^Pd and ^85^Rb, together with other elements (^10^B, ^111^Cd, ^161^Dy, ^151^Eu, ^69^Ga, ^7^Li, ^60^Ni, ^128^Te, ^203^Tl, ^169^Tm, ^51^V, ^171^Yb, and ^68^Zn) as significant markers to distinguish raw meat between countries [66].

### 3.5. Health Risk Assessment through Pork Meat Consumption

In this study, the mean values of metal concentrations were used to estimate the potential health risks associated with the consumption of pork meat via the calculation of EDI, THQ, HI, and TR. In order to evaluate the safety of the investigated pork meat samples coming from Romania and abroad (Germany, Spain, and Hungary), with respect to their metal concentrations (As, Cd, Pb, Sn, Cu, and Zn), the daily intake of elements was calculated from meat consumption and compared with the provisional tolerable daily intake (PTDI) for humans (Table 3). In relation to the non-carcinogenic risk factor of metals, the parameters (THQ and HI) were calculated, and the values of these parameters are indicated in Table 4. TR is a tool used to assess the cancer risk of analyzed metals. The U.S. Environmental Protection Agency accepted for regulatory purposes a cancer risk in the range of 1 × 10^−6^ to 1 × 10^−4^ [68]. TR values are presented in Table 5.

As shown in Table 3, the EDI values (μg/kg bw) obtained in the present study ranged from 0.048 to 0.097 (As), 0.022 to 0.058 (Cd), 0.115 to 0.253 (Sn), 0.079 to 0.168 (Pb), 1.317 to 1.926 (Cu), and 10.162 to 18.099 (Cu), respectively. These were significantly lower contents than the PTDI for As, Cd, Pb, Cu, and Zn, which was 2.14 μg/kg bw/day; 0.8 μg/kg bw/day; 3.57 μg/kg bw/day; 500 μg/kg bw/day, and 1000 μg/kg bw/day, respectively, which means that human consumption of investigated meat samples with such metals had a minimum health risk.

THQ and HI results were far lower (Table 4), which suggests a minimal risk of non-carcinogenic consequence per metal for consumers that eat the investigated pork meat samples. TR values were within the guideline value, which implies that none of the analyzed metals in the present study pose a carcinogenic risk (Table 5).

## 4. Conclusions

In this study, a total set of 70 loin pork samples were collected, and isotopic signatures (δ^2^H, δ^18^O, and δ^13^C) and 29 elements were determined. The multivariate statistical approaches (ANOVA, PCA, and LDA) were applied to obtain the most important predictors to distinguish the meat samples’ geographical origin: Romania versus abroad (Spain, Germany, and Hungary). The three components retained explained the 45.7% variability. PC1 explained 24.4% of the variability based on As, Mo, Cd, Sb, La, and Pb, having a positive contribution on PC1, and PC2 explained the additional 11.5% of variability where Ce, In, and Pd had a positive correlation on PC2. The third principal component, PC3, contributed to 9.8% of the variance based on K, Na, and Mg and could represent the geological influence upon sample distribution. By applying the LDA model, 91.4% was obtained in the initial classification, while for the cross-validation procedure, 90.0% was recorded. δ^2^H, K, Rb, and Pd were the most representative markers identified according to the meat samples’ geographical origin.

A human health risk assessment through pork meat consumption was realized, taking into account As, Cd, Sn, Pb, Cu, and Zn concentrations. THQ and HI results were far below 1, suggesting a minimal risk of non-carcinogenic consequence per metal for consumers eating the investigated pork meat samples. TR values were within the guideline values, which assumes that none of the analyzed metals in the present study pose a carcinogenic risk.

Further study will be developed by increasing the number of meat samples and corroborating IRMS and ICP-MS techniques with other supervised chemometric methods, not only for geographical origin attribution of samples but also for swine diet identification.

## Figures and Tables

**Table 1:** Concentration levels of macro, micro, and trace elements in pork meat samples.

Elements	Domestic	Imported		
	Romania	Spain	Germany	Hungary
	Concentration (g/kg)			
**Mg**	0.24 ± 0.04	0.25 ± 0.10	0.25 ± 0.02	0.27 ± 0.02
**Ca**	0.07 ± 0.11	0.04 ± 0.03	0.05 ± 0.03	0.06 ± 0.02
**Na**	0.51 ± 0.15	0.47 ± 0.13	0.44 ± 0.13	0.55 ± 0.07
**K**	4.21 ± 0.90	4.51 ± 1.14	4.10 ± 0.69	4.63 ± 0.51
**Concentration (mg/kg)**				
**B**	0.51 ± 0.30	0.48 ± 0.30	0.73 ± 0.25	0.57 ± 0.20
Cr *	0.42 ± 0.17	0.52 ± 0.21	0.70 ± 0.28	0.38 ± 0.16
**Mn**	0.09 ± 0.08	0.09 ± 0.04	0.09 ± 0.02	0.09 ± 0.03
**Fe**	6.63 ± 4.82	8.35 ± 4.85	6.40 ± 2.49	9.15 ± 8.73
Ni *	0.11 ± 0.07	0.19 ± 0.19	0.57 ± 0.98	0.10 ± 0.03
**Cu**	0.94 ± 0.49	0.76 ± 0.57	1.12 ± 0.27	0.61 ± 0.40
**Zn**	7.26 ± 2.63	8.80 ± 3.65	7.45 ± 1.71	6.91 ± 2.13
**Rb**	3.71 ± 1.03	3.24 ± 1.15	2.90 ± 0.53	3.07 ± 0.92
**Ba**	0.11 ± 0.14	0.22 ± 0.22	0.19 ± 0.23	0.07 ± 0.01
**Pd**	0.08 ± 0.05	0.08 ± 0.05	0.06 ± 0.03	0.09 ± 0.05
**Li**	0.03 ± 0.02	0.04 ± 0.02	0.02 ± 0.02	0.04 ± 0.01
**V**	0.04 ± 0.02	0.04 ± 0.02	0.05 ± 0.03	0.04 ± 0.03
**Co**	0.01 ± 0.02	0.01 ± 0.01	0.01 ± 0.01	0.01 ± 0.01
**Sr**	0.03 ± 0.03	0.03 ± 0.01	0.03 ± 0.01	0.02 ± 0.00
**Mo**	0.03 ± 0.02	0.04 ± 0.03	0.03 ± 0.03	0.03 ± 0.02
**As**	0.03 ± 0.03	0.03 ± 0.03	0.03 ± 0.04	0.06 ± 0.04
**Cd**	0.03 ± 0.03	0.03 ± 0.03	0.01 ± 0.03	0.03 ± 0.03
**In**	0.02 ± 0.03	0.03 ± 0.02	0.01 ± 0.02	0.04 ± 0.03
**Sn**	0.08 ± 0.04	0.12 ± 0.20	0.14 ± 0.14	0.08 ± 0.03
**Sb**	0.02 ± 0.02	0.03 ± 0.02	0.01 ± 0.02	0.03 ± 0.03
**La**	0.01 ± 0.01	0.01 ± 0.01	0.004 ± 0.01	0.01 ± 0.01
**Ce**	0.004 ± 0.004	0.01 ± 0.003	0.003 ± 0.002	0.01 ± 0.004
**Gd**	0.0004 ± 0.0002	0.0004 ± 0.0003	0.0005 ± 0.0002	0.0004 ± 0.0003
**Tl**	0.01 ± 0.02	0.01 ± 0.02	0.02 ± 0.03	0.02 ± 0.03
**Pb**	0.07 ± 0.06	0.08 ± 0.05	0.05 ± 0.04	0.08 ± 0.04

* Statistically significant differences among meat samples from different geographical origins (*p* = 0.008 for Ni, *p* = 0.015 for Cr).

**Table 2:** Classification results, ^c^ based on the LDA model.

		1-Ro 2-Foreign	Predicted Group Membership		Total
			Romania	Foreign	
**Original**	Count	1.00	34	3	37
		2.00	3	30	33
	%	1.00	91.9	8.1	100.0
		2.00	9.1	90.9	100.0
Cross-validated *	Count	1.00	34	3	37
		2.00	4	29	33
	%	1.00	91.9	8.1	100.0
		2.00	12.1	87.9	100.0

* Cross validation is conducted only for those cases in the analysis. In cross validation, each case is classified by the functions derived from all cases other than that case.

**Table 3:** Estimated daily intake (EDI) (μg/kg bw) of metals in pork meat samples from Romanian supermarkets.

Metal	EDI (μg/kg bw) in Investigated Pork Meat				
	Romania (n = 37)	Spain (n = 25)	Germany (n = 5)	Hungary (n = 3)	PTDI
**As ^1^**	0.048	0.070	0.056	0.097	2.14
**Cd ^1^**	0.036	0.058	0.022	0.047	0.8
**Sn**	0.115	0.253	0.233	0.181	2.0
**Pb ^2B^**	0.100	0.168	0.079	0.138	3.57
**Cu ^3^**	1.317	1.558	1.926	1.381	500
**Zn ^3^**	10.162	18.099	12.775	13.182	1000

n—the number of investigated samples. Provisional tolerable daily intake value (μg/kg bw/day) of metals established by the Joint FAO/WHO Expert Committee on Food Additives (JECFA). The International Agency for Research on Cancer (IARC) classification for metals: Group 1—carcinogenic to humans; Group 2B—possibly carcinogenic to humans; Group 3—not classifiable as to its carcinogenicity to humans.

**Table 4:** Target hazard quotient (THQ) and non-carcinogenic (HI) risk in pork meat samples.

Origin	Estimated THQ in Pork Meat Samples × 10^−3^						HI × 10^−3^
	As	Cd	Sn	Pb	Cu	Zn	
Romania (n = 37)	0.1590	0.0361	0.0002	0.0249	0.0329	0.0339	0.2870
Spain (n = 25)	0.2340	0.0584	0.0004	0.0420	0.0389	0.0603	0.4341
Germany (n = 5)	0.1852	0.0216	0.0004	0.0198	0.0482	0.0426	0.3178
Hungary (n = 3)	0.3221	0.0474	0.0003	0.0346	0.0345	0.0439	0.4828

**Table 5:** Target cancer risk (TR) of metals in investigated pork meat samples.

Metal	Estimated TR			
	Romania (n = 37)	Spain (n = 25)	Germany (n = 5)	Hungary (n = 3)
As	7.15 × 10^−8^	1.05 × 10^−7^	8.33 × 10^−8^	1.44 × 10^−7^
Cd	1.37 × 10^−8^	2.22 × 10^−8^	8.20 × 10^−9^	1.80 × 10^−8^
Pb	8.46 × 10^−10^	1.42 × 10^−9^	6.73 × 10^−10^	1.17 × 10^−9^
Cu	1.97 × 10^−6^	2.33 × 10^−6^	2.88 × 10^−6^	2.07 × 10^−6^
Zn	3.04 × 10^−6^	5.42 × 10^−6^	3.83 × 10^−6^	3.95 × 10^−6^

## Data Availability

Data are contained within the article.
